# Comparison of the Thermal Properties of Geopolymer and Modified Gypsum

**DOI:** 10.3390/polym13081220

**Published:** 2021-04-09

**Authors:** Karol Prałat, Justyna Ciemnicka, Artur Koper, Katarzyna Ewa Buczkowska, Piotr Łoś

**Affiliations:** 1 Faculty of Civil Engineering, Mechanics and Petrochemistry, Institute of Building, Warsaw University of Technology, I. Łukasiewicza 17, 09-400 Płock, Poland; Justyna.Ciemnicka@pw.edu.pl (J.C.); Artur.Koper@pw.edu.pl (A.K.); 2 Department of Material Science, Faculty of Mechanical Engineering, Technical University of Liberec, Studentska 2, 461-17 Liberec, Czech Republic; Katarzyna.Ewa.Buczkowska@tul.cz (K.E.B.); Piotr.Los@tul.cz (P.Ł.); 3 Department of Materials Technology and Production Systems, Faculty of Mechanical Engineering, Lodz University of Technology, Stefanowskiego 1/15, 90-001 Lodz, Poland

**Keywords:** thermal properties, thermal conductivity, micro additives, geopolymer, gypsum

## Abstract

The paper presents the results of research concerning the influence of micromaterials on the heat conductivity coefficient *λ*, specifically heat *Cp* and thermal diffusivity *a* of modified gypsum and geopolymer. Microspheres, hydroxyethyl methylcellulose (HEMC) polymer, and aerogel were used as the gypsum’s modifying materials. The study also investigated an alkali potassium-activated methakaolin-based geopolymer with the addition of aluminium dust. During the measurements of thermal parameters, the nonstationary method was chosen, and an Isomet device—which recorded the required physical quantities—was used. When compared to the reference sample, a decrease in the thermal conductivity and diffusivity of the hardened gypsum— and a simultaneous increase in specific heat—was observed with the addition of micromaterials. The geopolymer sample was characterized by the lowest value of thermal conductivity, equal to 0.1141 W/(m·K). It was over 62% lower than the reference sample containing only gypsum. The experimental values of the thermal conductivity of the gypsum samples with the addition of HEMC, aerogel and microspheres were, respectively, over 23%, 6%, and 8% lower than those of the unmodified gypsum samples. The lowest values of thermal conductivity were observed in the case of the gypsum samples modified with polymer; this resulted from the fact that the polymer caused the greatest change in the structure of the gypsum’s composite, which were expressed by the lowest density and highest porosity.

## 1. Introduction

Sustainable development in the construction industry is associated with special care for the environment at every stage of the construction process. One sector that is very important for sustainable development today is the energy industry—especially energy conservation. Material engineering fits very well into these areas. Innovative building materials that have good thermal and strength properties are constantly being sought out. The thermal insulation properties of materials can significantly contribute to the improvement of the functioning of buildings, the reduction of heat losses, lower energy consumption and, consequently, lower CO_2_ emissions. All of this contributes to environmental protection goals. Regarding sustainable development and building materials, solutions concerning multifunctional mortars are desirable [1]. Such materials can combine the properties and functions of different mortars. An example materials type worth noting is heat-insulating gypsum mortars. Apart from their good mechanical properties, these mortars are also characterized by good thermal properties, including especially low values of the thermal conductivity coefficient *λ*. Polymers are very often used as additives in building materials, and—when embedded in the structure of substances—they enable composites with new thermal properties to be obtained.

Various types of polymers are used in the production of innovative building materials. There are studies in the literature concerning the influence of polymer additives on concrete [2] and cement [3,4]. In these studies, the applied inorganic polymers reduced the value of thermal conductivity, while simultaneously increasing the porosity of samples. Heim et al. [5] made an attempt to determine the effect of hydroxyethyl methyl cellulose (HEMC) on the thermal conductivity of gypsum. The authors tested composites containing 0.1% and 1% of polymer in a sample. It was shown that the addition of 1% of polymer to the gypsum samples caused a decrease in thermal conductivity of over 20% (within the range of 0.295–0.355 W/(m·K)) when compared to the reference samples without the addition of HEMC. The authors of the publication stated that, in order to fully determine the effect of the additive on the thermal properties of composites, further studies would need to be carried out with different amounts of polymer in the samples, with different mass ratios of water to gypsum.

The authors of paper [6] showed that a small addition of methylcellulose (up to 0.4%) to a gypsum sample had a significant impact on the physical properties and microstructure of mortars. However, the article did not conduct thermal studies of modified gypsum composites. In turn, the influence of the viscosity of an aqueous solution of hydroxypropyl methylcellulose (HPMC) on the selected physical properties of modified gypsum samples was considered in paper [1]. The polymer significantly influenced the microstructure of the tested samples. Mortars without the addition of HPMC had very well-developed crystalline forms of gypsum. In contrast, samples with polymer additives were characterized by much smaller crystals, with clearly marked membranes in the pores of the composite. It should be assumed, then, that the internal structure of building materials obtained in this way will significantly affect the thermal properties of samples. The authors of the study, however, did not undertake such analyses.

The addition of organosilicon compounds to cement- and gypsum-based mortars was described in paper [7]. The influence of silicate additives on setting time, water absorption, porosity, bending/compressive strength, and frost resistance was tested. The studies showed that the addition of polydimethylsiloxane had a particularly beneficial effect on the microstructure of composites. In the above-mentioned studies [1,6,7], apart from conducting interesting microscopic and strength tests, the authors did not analyse the thermal properties of the obtained modified materials.

The authors of this paper noticed a significant lack of information concerning the influence of many additives on the thermal properties of modified gypsums. The observed shortcomings necessitated the conduction of research related to the use of various additives, including hydroxyethyl methylcellulose polymer, in order to analyze the thermal properties of modified building materials. The article presents the results of measurements of the thermal conductivity *λ*, specific heat *C_p_*, thermal diffusivity *a*, and bulk density *ρ_b_* of the obtained gypsum composites. In addition, apart from the results concerning gypsum materials, the thermal properties of foamed geopolymers were also analyzed, summarized and compared. Geopolymer materials have become more crucial construction technologies in recent years.

The aim of the current study was to juxtapose and compare the influence of various gypsum additives, which differ in terms of their chemical structure (polymer, microspheres, aerogel) and the thermal parameters of the obtained composites. A summary of different groups of additives which focused on the thermal properties of materials could not be found in the literature. Gypsum materials, unlike cement materials, are a slightly forgotten building component; therefore it is important to investigate innovative, modified gypsum composites and indicate their real insulation advantages. Nowadays, geopolymers are an interesting material, thought they are still being studied. This paper studied these two groups of materials in response to growing interest in insulated building materials. The paper presents measurements of three thermal parameters: thermal conductivity, specific heat and thermal diffusivity, which are often considered in separate publications.

## 2. Materials and Methods

### 2.1. Materials

The geopolymer material used was an alkali potassium-activated double-component aluminosilicate binder based on metakaolin, produced industrially by Baucis LK (České Lupkové Závody, Pecinov, Czech Republic) [8]. In order to create pores inside the geopolymer, aluminum powder (Pkchemie Inc., Třebič, Czech Republic) was added. The dust contained 99% aluminum with an average grain size of 65 µm [9]. The mass ratio of the aluminum powder to the aluminosilicate binder with an activator was 0.5%. At the same time, according to the manufacturer’s guidelines, the activator was added to the aluminosilicate powder in a mass ratio of 0.9.

The foamed geopolymer samples were made according to the following steps. First, the geopolymeric binder was combined with the potassium activator, in the ratio recommended by the manufacturer. Then, the ingredients were mixed for 5 min at room temperature until a homogeneous mixture was obtained. The aluminum powder was added at the end of the product’s manufacturing stage, after which the entire sample was mixed vigorously in order to foam the geopolymer and create pores. The obtained product was placed in 15 × 15 × 15 cm^3^ molds, then left for two hours to produce a geopolymer foam. Next, the geopolymer samples were cured overnight at 70 °C; the gypsum samples were conditioned at 20–22 °C at a humidity of 52–54% for 28 days in the hygrothermal conditions prevailing in the laboratory. Based on the experience of other authors [10,11,12,13], measurements of the thermal properties of the materials were taken after 28 days of conditioning.

At the same time, in order to compare the thermal properties of the obtained geopolymers, samples made of modified gypsum—based on building gypsum (Nida Valley, Pińczów, Poland)—were prepared. The additives used to modify the properties of the reference sample included hydroxyethyl methylcellulose (HEMC) polymer, microspheres and aerogel.

Among the many cellulose ethers, HEMC (Sigma Aldrich, Warsaw, Poland) has found a wide application in material engineering, as it is used to modify building materials based on gypsum or cement [5,14,15,16]. In turn, silica aerogel particles were used; the particles had a fraction size within the range of 0.7–4.0 mm [17,18]. This material is characterized by a very high porosity, which is expressed by a low specific density ranging from 130–150 kg/m^3^. Due to these properties, this material—which has nanostructured pores with a diameter of about 20 nm—has a thermal conductivity of only 0.012–0.018 W/(m·K) [19]. The aerogel used in the study was a translucent material manufactured by Cabot Corporation (Leuven, Belgium). The third additive used to modify the gypsum was a microsphere material, manufactured by Eko Export Inc. (Bielsko-Biala, Poland). The material was comprised of light, thin-walled spheres with diameters ranging from 50 to 150 μm. They originated from combined heat and power plants, in which they were created as by-products of pulverized coal combustion. There are already known cases of using these waste materials in the production of composites based on cement [20,21,22,23] and gypsum [24,25,26,27]. The main chemical components of the microspheres were about 35% aluminum oxide Al_2_O_3_, and about 55% silicon oxide SiO_2_.

**Figure 1:**
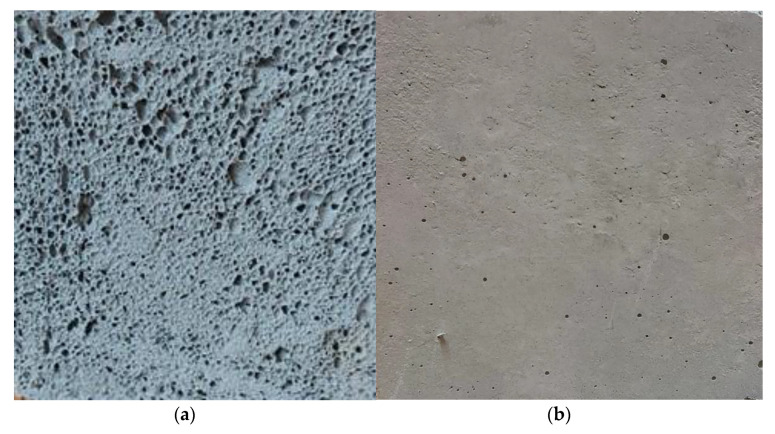
Samples used in thermal tests after 28 days of conditioning in heat/humid conditions: (**a**) geopolymer, (**b**) gypsum.

The modified gypsum samples were made of building gypsum, mixed first with polymer, aerogel or microspheres in the appropriate mass ratio. After the ingredients were thoroughly mixed, water was added with a constant ratio to gypsum of w/g = 0.75. HEMC and aerogel were added at 1% concentration; microspheres were added at 10% concentration with regards to the weight of the gypsum. All the gypsum and geopolymer slurry recipes are included in Table 1. The compounds of the modified gypsum were mixed for 1 min at 20 °C and then placed in identical forms to the geopolymer samples. The gypsum composites were conditioned for 28 days under the same conditions as the geopolymer samples. The view of the geopolymer and gypsum samples after the conditioning time is shown in Figure 1a,b.

More detailed information concerning the chemical structure, chemical composition and microstructure of the additives used is presented in publications [5,14,15,16].

### 2.2. Methods

The thermal parameters (thermal conductivity *λ*, volumetric thermal capacity *C_v_*, and thermal diffusivity *a*) of the obtained samples were tested using the Isomet 2114 device after 28 days of incubation.

**Figure 2:**
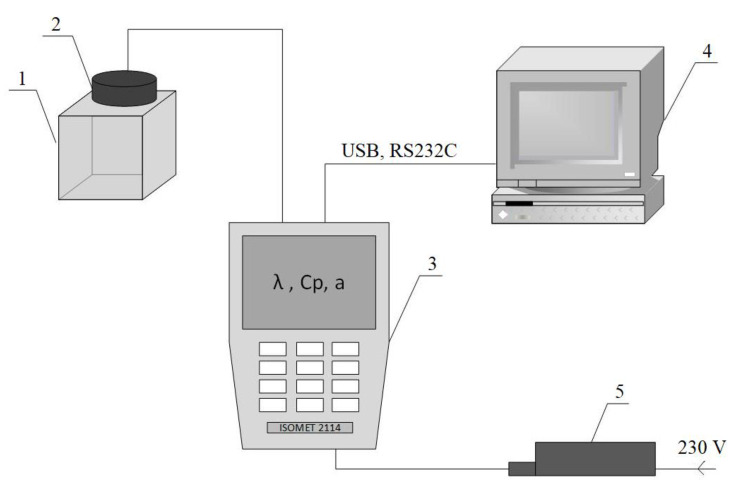
Scheme of the experimental stand for measuring the thermal properties of building materials [24,29]: 1—test sample, 2—probe, 3—Isomet 2114 device, 4—computer, 5—power supply.

The measurement method was based on measurements conducted in nonstationary conditions. Measurement methods based on undetermined heat conduction often result in the determination of thermal diffusivity on the basis of measuring the change in temperature during the heating or cooling of a sample. A measurement that does not require a determined heat flow can be performed using the proposed measuring stand. The device analyses’ temperature changes resulted from the response of the tested material to the flow of thermal impulses. These changes were measured using changeable probes, which were connected with a meter attached to a computer that recorded the results (Figure 2). During the measurement process, the amount of heat generated by the device was known. Heat propagated radially in the sample. The increase in the temperature of the sample changed linearly with the logarithm of time. This relationship allowed the thermal conductivity of the tested material to be directly obtained [24,28].

**Figure 3:**
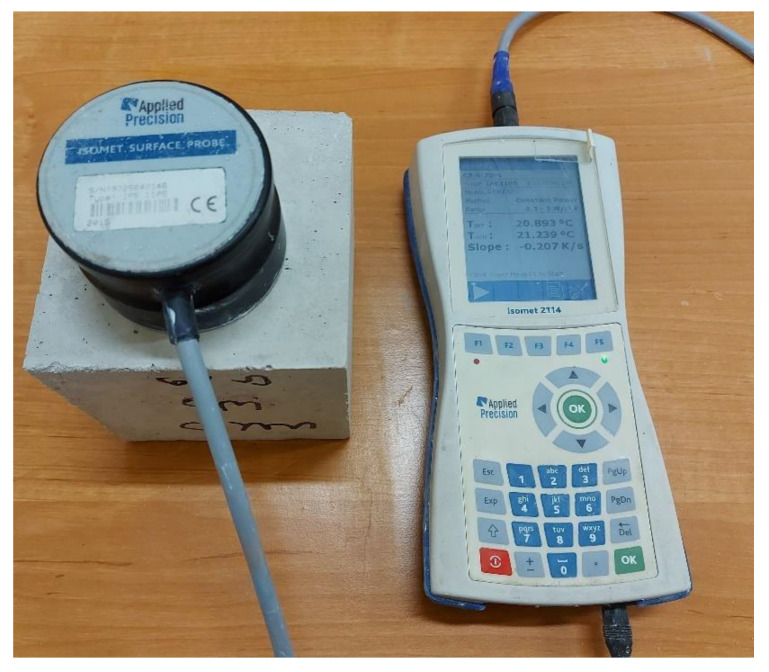
Investigations of the thermal properties of the modified building samples.

The device used features a wide measuring range and can be used, among other things, for insulation, building materials, plastics, glass, and minerals. The measuring range depends on the probe used, and covers *λ* values from 0.015 to 6.0 W/(m·K) and *C_v_* values from 0.04 to 3 MJ/(m^3^·K). The meter has two optional types of probes: needle probes for soft materials, and surface probes for hard materials. Measurement data can be saved in the internal memory of the device or exported to a computer. In the presented experiment, the measurements were conducted using a surface probe (Figure 3).

## 3. Results and Discussion

### 3.1. The Results of Density and Porosity

All the samples were accurately weighed after 28 days of incubation. Because their dimensions and mass were known, the volumetric density of the samples *ρ_b_*_1_ was calculated using simple relationship Equation (1):

(1) $$\rho_{b1}=\frac{m}{V}$$

In addition, the porosity of the samples was determined on the basis of the specific density of the gypsum and geopolymer. Based on the measured thermal parameters (described in Section 3.2), it was possible to calculate the density *ρ_b_*_2_ from Equation (6). Both of the density values are summarized in Table 2. The obtained density values of all the samples *ρ_b_*_1_ and *ρ_b_*_2_ did not differ by more than 1.5%. The smallest differences (0.11%) were obtained for the polymer-modified (GP) gypsum sample. Moreover, in the case of the geopolymer (GF) sample, the calculated differences were 0.36%. The calculated densities of *ρ_b_*_1_ and *ρ_b_*_2_ did not differ significantly, despite the use of different calculation methods. In the first case, the density was calculated based on the known masses and volumes of the samples, and in the second case based on their thermal properties.

It was noted that the gypsum-modifying additives reduced the density by 6–8%; at the same time, the porosity increased by 4–5% when compared to the reference sample that contained only pure gypsum. The geopolymer (GF) sample was characterized by a very low density, and a very high porosity. The pores formed during the geopolymer manufacturing process can be seen in Figure 1a.

### 3.2. The Results of the Thermal Properties and Their Discussion

The thermal measurements of all five samples were conducted, and the following were obtained: thermal conductivity *λ*, volumetric heat capacity *C_v_*, and thermal diffusivity *a*. Six measurement series were performed each time. The measurement results with the obtained statistical data are presented in Table 3. The specific heat *C_p_* expressed in J/(kg·K) was obtained by dividing the measured volumetric heat capacity *C_v_* by the material’s volume density *ρ_b_*_1_.

If the standard deviation of the random variable *X* is unknown, the distribution of the arithmetic mean of sample $\bar{X}$ is very well approximated by the Student’s t-distribution. The Student’s random variable is defined as follows Equation (2):

(2) $$t=\frac{\bar{X}-\mu }{s},$$

where: *s*—the standard deviation of the sample, *µ*—the expected value.

A probability density function of this random variable is expressed using Equation (3):

(3) $$f(t,n)=\frac{\Gamma (\frac{n+1}{2})}{\Gamma (\frac{n}{2})\sqrt{n\pi }}{\left(1+\frac{t^{2}}{n}\right)}^{-\frac{n+1}{2}},$$

where *Γ*(*x*) is the Euler’s gamma function.

The detailed form of the probability density function Equation (2) depends on the number of degrees of freedom (*n* − 1). The graph of this function is symmetrical in relation to *t* = 0, and the smaller the number of degrees of freedom (*n* − 1), the more flattened it gets. The fewer observations (measurements) that are conducted for the purpose of calculating the mean value $\bar{X}$, the more this value will deviate from the real (expected) value of the random variable *X*. Of course, with an increase in the number of degrees of freedom, the Student’s t-distribution tends toward normal distribution *N*(0, 1).

If the tested random variable had the *N(µ*, *σ)* distribution, and the standard deviation is unknown, we built the confidence interval using the Student’s t-distribution with the probability density expressed by Equation (3). We then obtained dependence Equation (4):

(4) $$P(-t_{n-1;\;\alpha /2}\leq \frac{\bar{X}-\mu }{s}\sqrt{n}\leq t_{n-1;\;\alpha /2})=1-\alpha .$$

After simple transformations, we finally received dependence Equation (5):

(5) $$P(\bar{X}-t_{n-1;\;\alpha /2}\frac{s}{\sqrt{n}}\leq \mu \leq \bar{X}+t_{n-1;\;\alpha /2}\frac{s}{\sqrt{n}})=1-\alpha ,$$

where *α* is the assumed significance level, and 1 − *α* is the confidence level.

With the results of *n* measurements, the parameters—such as mean value $\bar{X}$, and also standard deviation *s*, calculated from the sample—were determined. The intervals of the actual measured values—thermal conductivity *λ*, specific heat *C_p_*, and thermal diffusivity *a*—were estimated with a certain probability. For all the performed measurements, the measurement uncertainty was assessed based on Student’s t-distribution. Based on statistical calculations, and with the assumed confidence level of 95%, the confidence intervals of the measured thermal properties were determined. With a probability close to one, the sought values of the thermal parameters (*λ*, *C_v_*, *a*) of the geopolymer and gypsum samples are within the intervals shown in Table 4.

The geopolymer sample was characterized by the lowest value of thermal conductivity, equal to 0.1141 W/(m·K). It was over 62% lower than that of the reference sample, which contained pure gypsum. In papers [30,31], the authors also used aluminum dust as a type of foam agent. The researchers obtained thermal conductivity of the foamed geopolymers at 0.12 W/(m·K) in the case of a density of 0.36 kg/m^3^ [30], and 0.10 W/(m·K) in the case of a density of 0.55 kg/m^3^ [31]. The authors of paper [32] improved the thermal conductivity of geopolymer by simultaneously foaming it and introducing a hollow glass bead (HGB) into it. The products received in this way had a low density, and at the same time, a low value of the thermal conductivity coefficient. The obtained composites were characterized by densities of 300 kg/m^3^ and 250 kg/m^3^. The corresponding values of thermal conductivity were 0.0711 W/(m·K) and 0.0522 W/(m·K). In addition, the authors of the paper obtained composites with the ash of rice husk, silica, and slag; these composites had values of conductivity λ ranging from 0.170 to 0.353 W/(m·K). These values were then compared to the existing thermal-insulation gypsum with λ = 0.170–353 W/(m·K).

In article [10], the influences of mixing parameters, foaming agent and surface-active agent on the physical and mechanical properties (as well as on the thermal conductivity) of a metakaolingeopolymer were investigated. The introduction of H_2_O_2_ and a surface-active agent reduced the compressive strength (0.4–6.0 MPa), bulk density (471–1212 kg/m^3^), and thermal conductivity to values ranging from 0.11 to 0.30 W/m·K—and at the same time, increased the porosity (36–86%). The increased pore volume resulted from the simultaneous use of H_2_O_2_ and the surface-active agent. The surface-active agent also aided the aeration of the samples and acted as a pore stabilizer. Paper [33] presented the results of tests of lightweight cement composites with empty glass microspheres. It was shown that the chemical stability of the microspheres could be directly controlled by the modulation of the specific surface area and constant dissolution rate of supplemental silica additives. In addition to their stabilizing effect, these additives led to an improvement in the pores’ structure. The obtained composites were characterized by a value of thermal conductivity below 0.3 W/(m·K). This class of composites has the potential to improve insulation and increase the energy efficiency of external partitions.

The differences in the obtained values of thermal conductivity probably resulted from the different amounts of water in the samples, and also from the insufficiently dried composites. Additionally, the thermal properties of geopolymers relate to both the geopolymers’ components and the raw materials used. When looking for materials with very good thermal insulation properties, foamed porous geopolymer materials are a very good alternative to cement binding materials. Currently, innovative geopolymer materials are produced by incorporating solid aluminum/silicon components, liquid activators, foaming agents or porous additives into their structure. All the discussed test results should be treated individually, as different materials and additives were used.

The experimental values of the thermal conductivity of the gypsum samples with the addition of HEMC polymer, aerogel and microspheres were lower than those for the unmodified gypsum samples by over 23%, 6% and 8%, respectively. The influence of the used HEMC polymer on the thermal conductivity of the gypsum was significant. It was obvious that, during the first days of hydration, the samples contained significant amounts of water which was not involved in the chemical reactions. The water contained in the samples evaporated during conditioning over 28 days, after which the samples reached the air-dry state. The used additives increased the porosity of the materials and decreased their density (Table 2). At the same time, the additives reduced the value of thermal conductivity *λ*. The addition of polymer caused the gypsum density to decrease by almost 6% when compared to the reference sample. Heim et al. [5] also noted that the amount of polymer used had a significant impact on density, porosity and thermal conductivity. They used cellulose derivatives at 0.1% and 1%, and showed that the density and thermal conductivity of the samples decreased with the addition of polymer, as the porosity increased. The polymer influenced the rate of the setting of the gypsum. It changed the structure of the composite, which could be seen in the physicochemical parameters of the modified product. The main differences in the physical parameters of the tested composites were noticed after 28 days of hydration (i.e., after full stabilization of the moisture content in a sample). The polymer was absorbed on the surface of the hemihydrate and dihydrate grains, which reduced the nucleation rate and increased the porosity. The polymer significantly changed the gypsum crystallization conditions. This action of the polymer adversely affected the strength of the gypsum binder, while improving thermal conductivity. In order to prevent the polymer from causing an excessive drop in the strength of the gypsum material, the addition of small amounts of substances that plasticize the slurry and modify the crystal structure could be used at the same time.

The gypsum sample with aerogel (GA) was characterized by the lowest value of specific heat *C_p_*, equal to 1523 J/(kg·K). This addition filled the pores of the modified composite, and therefore reduced this parameter by 2% when compared to the reference sample. The microspheres (spherical and hollow particles of fly ash) and HEMC polymer caused an increase in the porosity of the modified samples (GP and GM), and consequently caused an increase in specific heat by 2–3%. The highest specific heat value of 1683 J/(kg·K) was characteristic of the very porous geopolymeric (GF) sample, and was over 8% higher than the value for the unmodified gypsum sample (G).

The third examined parameter during the experiment was thermal diffusivity *a*. This parameter characterizes a material’s ability to transport heat within its volume. Its value is directly proportional to the thermal conductivity coefficient, and inversely proportional to the product of density and specific heat Equation (6):

(6) $$a=\frac{\lambda }{\rho_{b2}\cdot C_{p}}.$$

**Figure 4:**
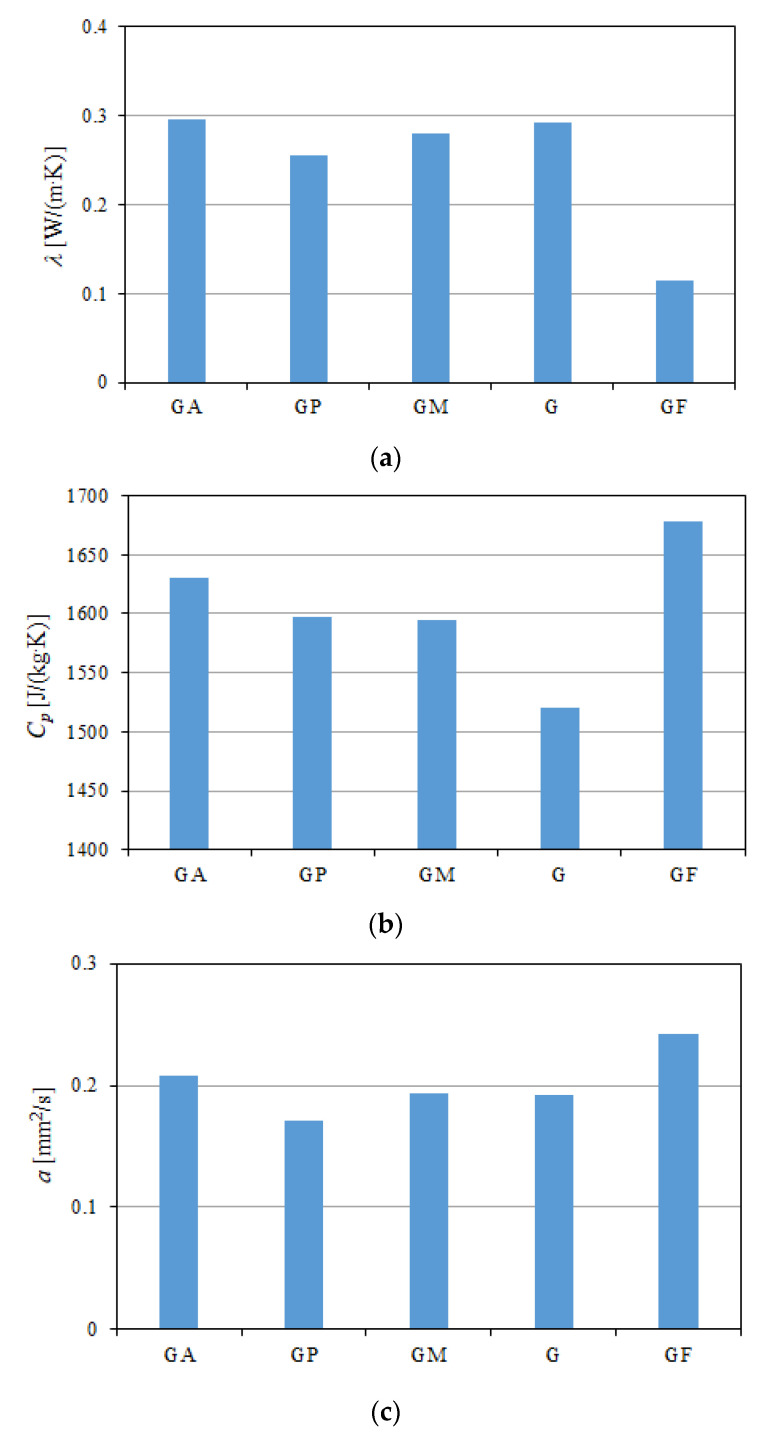
The obtained average values of (**a**) thermal conductivity, (**b**) specific heat, and (**c**) thermal diffusivity of modified gypsum samples and geopolymer.

The diffusivity coefficient determines the rate at which temperature changes from one plane to another, i.e., the susceptibility of a material to temperature equalization during heating or cooling if it has been subjected to a temporary thermal disturbance. Thermal diffusivity is a specific material property that characterizes heat conduction in undetermined conditions. This value allows for the determination of how quickly a material reacts to temperature changes. In order to be able to predict the behavior of a material during cooling and simulate spatial temperature changes, it is necessary to know the value of thermal diffusivity. This value is required when conducting calculations using the Fourier differential equation, which describes heat conduction in the undetermined state. The highest value of thermal diffusivity was obtained for the geopolymeric (GF) sample, and was almost 26% higher when compared to the gypsum sample (G). The lowest value was for the polymer-modified (GP) gypsum sample; it was 11% lower than that of the reference sample. The summarized values of the thermal conductivity *λ*, specific heat *C_p_* and thermal diffusivity *a* of all the tested samples are shown in Figure 4.

**Figure 5:**
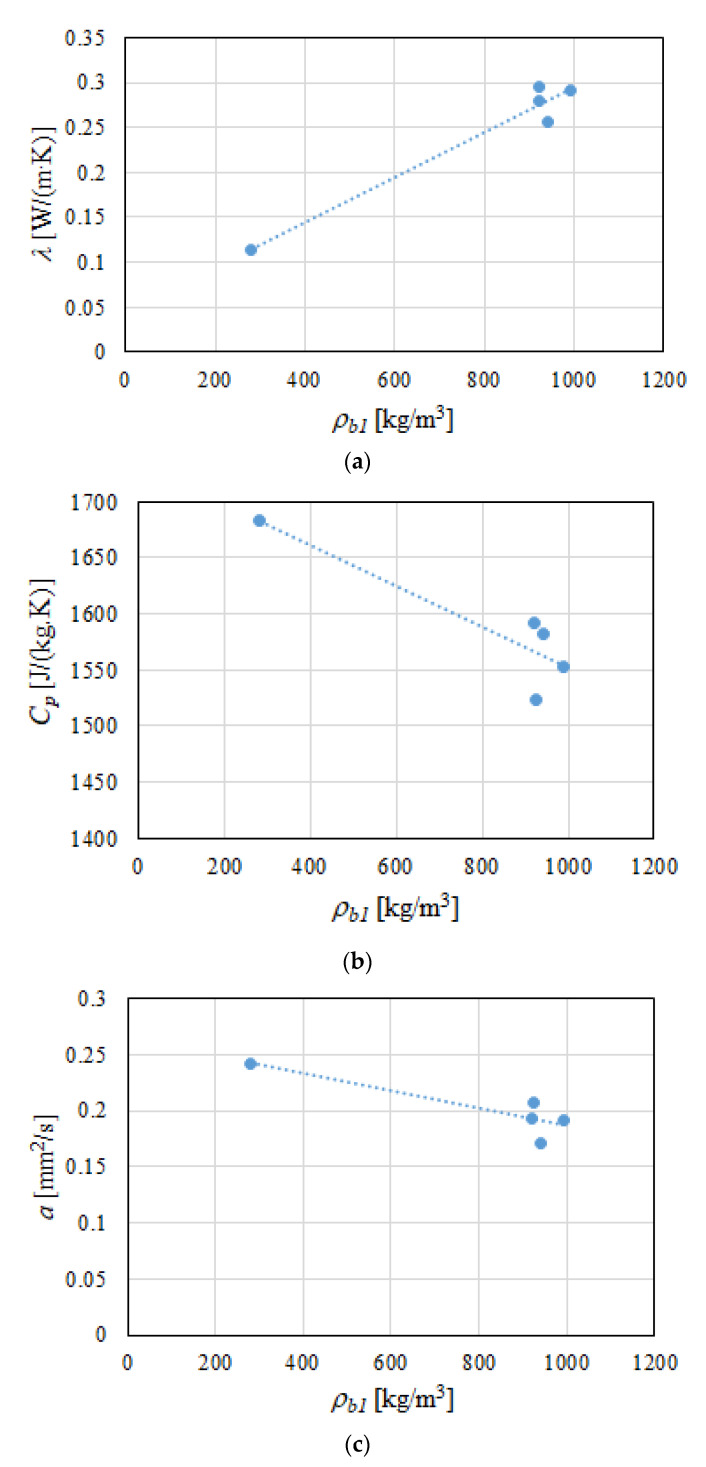
Graphs of the dependence of the (**a**) thermal conductivity, (**b**) specific heat, and (**c**) thermal diffusivity as a function of density for the modified gypsum samples and geopolymer.

Graphs of all the measured thermal properties in the samples’ density functions can be seen in Figure 5a–c. It was noted that, with the geopolymer density decreasing more than three times in relation to the density of the gypsum samples, the thermal conductivity *λ* also decreased (Figure 5a). At the same time, with the increase in the density of the modified gypsum samples, the values of specific heat *C_p_* (Figure 5b) and thermal diffusivity *a* (Figure 5c) decreased when compared to the geopolymer sample.

For the tested samples, dependencies Equations (7)–(9) were proposed; for the bulk density of the used building materials, they ranged from 281 kg/m^3^ to 991 kg/m^3^.

(7) λ=0.0002·ρ+0.0447

(8) Cp=−0.1806·ρ+1733.4

(9) a=−0.00008·ρ+0.2638

Due to the indirect method of determining thermal conductivity—based on the measurements of thermal diffusivity and volumetric heat capacity—nonstationary methods can provoke polemics and criticism among some researchers [34]. However, these measurements gained widespread recognition due to the short time required to complete single test. Nonstationary methods can obtain results in minutes, as compared to stationary methods, which can take several hours to do the same. Despite some objections, nonstationary methods are used for the thermal measurements of cement grouts [35], gypsum [5,18,36,37,38] and concrete modified with aggregates and waste materials [17,39,40,41]. The above-mentioned short summary of the frequent use of nonstationary measurements of thermal conductivity shows that these methods are growing in popularity, and that they are considered reliable. However, the authors of the present study point out that such tests should be carried out on samples in their dry state, especially when they have a porous structure.

When measuring the thermal conductivity of modified composite gypsum—where the value of the λ coefficient of water is more than twice than that of pure gypsum—it is particularly important to completely dry the samples. The presence of water in composite materials with a high porosity will cause the value of the tested parameter to be overestimated. Its presence may significantly distort the obtained measurement results.

**Figure 6:**
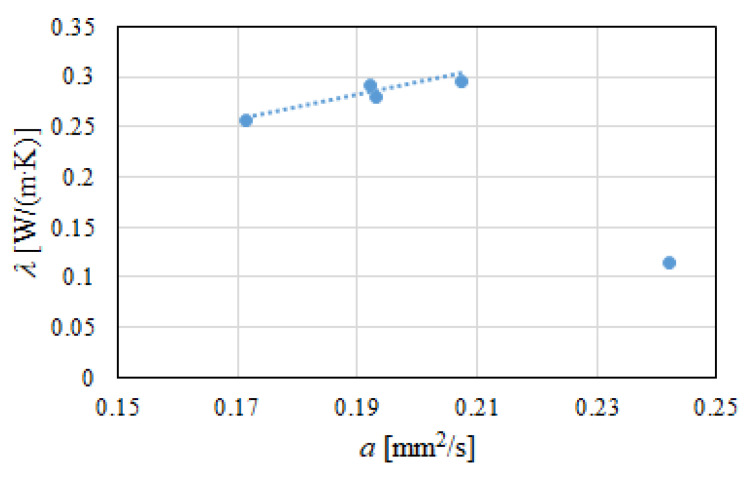
Graph of the dependence *λ* = *f*(*a*) for modified gypsum and geopolymer samples.

The performed thermal tests included the investigation of thermal conductivity *λ* under steady conditions and thermal diffusivity *a* under undetermined conditions. It is known that heat conduction is described by Fourier’s law. In such a situation, there is a linear temperature distribution in a function of the distance from the heated surface. In turn, in the case of an undetermined state, the tested sample is insulated on the side surface and supplied with heat that is evenly released on the front surface. Over time, the heat spreads along the sample, which warms. Of course, the temperature distribution along the sample is different depending on the time and place of measurement. Thermal diffusivity indicates the proportion between a material’s ability to conduct heat and its ability to heat up. Figure 6 shows a graph of the dependence of *λ* = *f*(*a*) for the modified gypsum and geopolymer samples.

The geopolymer (GF) sample showed the highest thermal diffusivity value, equal to 0.2421 mm^2^/s. In turn, among the tested gypsums, the highest values of *a* were found for the aerogel-modified sample (GA) and the reference sample (G); 0.2074 mm^2^/s and 0.1923 mm^2^/s, respectively.

In the case of the gypsum samples, a strong relationship was found between thermal conductivity and thermal diffusivity (Figure 6). For these samples, a linear relationship with a high correlation was observed and described by Equation (10). It was noticed that with an increase in diffusivity *a*, there was an increase in thermal conductivity *λ*. The relationship between these two parameters can be indirectly ascribed to the densities of the modified gypsum samples. The microspheres and the HEMC polymer that were used in the samples resulted in greater porosity, and thus a higher air content. At the same time, they reduced the values of thermal conductivity and heat transfer through the sample. Therefore, it was determined that various gypsum-modifying additives directly influenced the thermal properties of samples Equation (10):

(10) λ=1.1373·a+0.0633

There is a visible, growing demand for building materials that are environmentally friendly and fit within sustainable development initiatives. Innovative composites based on gypsum or geopolymers that have good thermal properties, including a low value of thermal conductivity *λ*, are constantly being sought.

The authors of paper [42] presented the thermal characteristics of Tunisian gypsum from the Meknassi region. The authors paid attention to the influence of temperature and moisture content on the values of thermal conductivity, whilst also obtaining values of *λ* within the range from 0.103 to 0.094 W/(m·K). The study of the thermal conductivity of gypsum boards was carried out by the authors of [43], who obtained values of *λ* = 0.19 W/(m·K) at 20 °C. Paper [44] also presented a summary of the values of thermal conductivity—obtained by many researchers—of gypsum boards. Obtained the results ranged from 0.18–0.4 W/(m·K). The authors of the study showed that the thermal conductivity of gypsum boards depended on their thickness, and also suggested that thin boards were better than thick boards at protecting steel structures under fire conditions. In turn, the average thermal conductivities of gypsum samples with wheat straw fiber content (of 0%, 1% and 3%) were 0.961, 0.596, and 0.310 W/m·K, respectively [45]. Article [46] presented the results of measurements of the thermal conductivity of gypsum materials containing capsules with a phase change material (PCM). The samples contained between 10% and 30% PCM, and the *λ* value was tested in a temperature range of 20–30 °C. The significant influence of temperature and the amount of PCM on the measured parameter was demonstrated. The increase in the amount of PCM in the sample—and also the increase in temperature—caused a decrease in the conductivity value, from 0.38 W/(m·K) to 0.08 W/(m·K). The PCMs in building partitions act as thermal energy storing elements, stabilizing temperatures in a given room and reducing the energy demand of buildings.

Various types of foaming agents can be used in the manufacturing of geopolymers. In this study, as well as in other studies [30,31,47,48], the authors used aluminum powder in order to foam the material and create pores. This powder reacts in a strongly alkaline environment and causes the release of hydrogen.

The authors of paper [47] obtained foamed geopolymers based on metakaolin, the thermal conductivity values of which were within the range of 0.12–0.35 W/(m·K). In turn, the researchers in [30] used rice husks and volcanic ash as additives to geopolymers, and obtained *λ* values from 0.12 to 0.17 W/(m·K). Paper [48] presented a study of the thermal behavior of metakaolin-based geopolymers that were foamed and reinforced with fibers. This reinforcement was meant to demonstrate the suitability of such composite materials as fireproof panels. During the experiment, the values of thermal conductivity within the range of 0.3–0.65 W/(m·K) were obtained. A very porous geopolymer material with fly ash additive was obtained in [31], and the thermal conductivity of this composite ranged from 0.1 W/(m·K) to 0.25 W/(m·K). In all publications, it was found that fine aluminum powders were a good additive for the geopolymerization process, as they resulted in light and porous structures with low thermal conductivity *λ*.

In addition to laboratory research, there are also important scientific studies related to the thermal conductivity of building materials, many based on theoretical modeling. From this perspective, theoretical fractal theory is a very important tool that can be used to study thermal conductivity. In article [49], the Fractal—Monte Carlo method was used to simulate the effective thermal conductivity of porous media with rough surfaces. The proposed probability model of the effective thermal conductivity of porous media with rough surfaces was expressed as a function of the relative roughness, porosity, minimum and maximum pore diameter, and fractal dimensions. The proposed model was verified by available experimental data. When analyzing the parameters of the microstructure of porous materials, the authors proved that the effective thermal conductivity of porous media with rough surfaces decreased with an increase in the fractal dimension and relative roughness. In addition, it was found that the proposed Fractal—Monte Carlo model could be used not only to model thermal parameters, but also to model the transfer of mass in porous media.

The authors of papers [50,51] presented a fractal analysis of the effective thermal conductivity of unsaturated fractal porous media, which was conducted on the basis of a thermoelectrical analogy and the statistical similarity of the porous media. The authors proposed a physical model of the thermal conductivity of a three-phase medium: solid-liquid-gas. When taking into account the porous characteristics of building materials, a coexisting three-phase model for calculating thermal conductivity—based on the capillary structure—was developed using the fractal theory. The quantitative influence of parameters—e.g., porosity, pore diameter distribution, moisture content and fractal structure—on the thermal conductivity of wet porous materials was also analyzed.

Taking into account the influence of a building materials’ pore size on heat transfer in the microscale of a material, the proposed model enabled the variability of thermal conductivity of pores (with regards to humidity) to be predicted. The pores of building materials are very small and mainly range from 0.01 mm to 10 mm. An analysis was made of the influence of the porosity, pore diameter distribution and parameters of the slotted structure on the thermal conductivity of wet porous materials by using the proposed model to calculate the thermal conductivity of moist porous building materials. Quantitiave moisture analysis was also conducted; it was found that with an increase in porosity, the rate of change in thermal conductivity first decreased and then increased. When the porosity was less than 0.1, this change was particularly visible.

During experiments, the authors of this publication obtained thermal conductivity values of λ = (0.2556–0.2959) W/(m·K) (Table 3) for the modified gypsum composites, and values of λ = 0.1141 W/(m·K) for the foamed geopolymer samples—which is close to the values received by many other authors [30,31,42,43,44,45,46,47,48]. The material investigations fall in line with global trends, which aim to search for innovative, environmentally friendly building materials with desired thermal properties. Modified geopolymer and gypsum materials are currently being investigated and of interest to many research centers around the world.

With regards to gypsum samples: in the future, it will be worth conducting research concerning the use of HEMC polymer in amounts other than those previously studied. It would also be worth checking the influence of other cellulose ethers on the mechanical and thermal properties of such composites. The preparation of geopolymers using other types of foam agents is an interesting area of study, and extensive studies of the various characteristics of such building materials would be valuable.

The parameters of innovative insulation materials (which are crucial from a thermal point of view) include: thermal conductivity, specific heat, and thermal diffusivity or thermal activity. Important mechanical parameters in the construction industry include: compressive and bending strength, air permeability and sorptivity, fire resistance, and acoustic properties. An interesting and unresolved issue involves investigations concerning plasticizing, air-entraining, foaming, loosening and sealing admixtures (as well as admixtures that delay and accelerate the setting of mortars) and their influence on the thermal and mechanical parameters of innovative building materials made using them. In order to better understand such materials, model studies should be carried out together with laboratory studies. Such joint coexistence would allow for practical (laboratory) knowledge to be complemented by theoretical (computer modeling) knowledge.

Sustainable development includes, among other things, care for the environment. In this context, research works should aim to increase the use of various types of wastes in construction materials, e.g., ash, slag, glass, solid polymers (including polyoxymethylene (POM)), sanitary ceramics, fibers and more. Newly formed composites have many unknown properties that need to be understood. Therefore, research should aim to better understand the effects of such additives on the physicochemical parameters of building materials, and also on human health.

## 4. Conclusions

It was found that the addition of hydroxyethyl methylcellulose polymer influenced water storage and the gypsum setting rate. It also changed the morphological structure of the gypsum-polymer composite, which could be seen in the density and porosity of the obtained product.

The calculated densities *ρ_b_*_1_ and *ρ_b_*_2_ of the obtained gypsum composites and the geopolymer did not differ significantly, despite the use of different calculation methods. In the first case, the density was calculated based on the known masses and volumes of the samples; in the second case, it was based on their thermal properties.

The lowest value of thermal conductivity *λ* was obtained in the case of the geopolymer samples. The value of thermal conductivity—0.1141 W/(m·K)—was 62% lower than for the reference sample with gypsum.

Among the additives used in this study, the addition of HEMC had the greatest impact on the thermal conductivity of gypsum. It caused a reduction of the *λ* coefficient by more than 23% compared to the reference sample. During the tests, a decrease in thermal diffusivity and an increase in the specific heat of the samples containing the used additives were also observed.

## Figures and Tables

**Table 1:** Composition of the geopolymer pastes and modified gypsums used in the research.

Tested Sample	Geopolymer/Gypsum [g]	Activator/Water [g]	Additive [g]
Geopolymer + aluminum powder (GF)	527	473	5.0
Gypsum (G)	572	428	-
Gypsum + Polymer (GP)	572	428	5.7
Gypsum + Aerogel (GA)	572	428	5.7
Gypsum + Microspheres (GM)	572	428	57.2

**Table 2:** The calculated bulk density and porosity of the tested samples.

Parameters	Tested Sample				
	GF	G	GP	GA	GM
Bulk density *ρ_b_*_1_ [kg/m^3^] Calculated from Equation (1)	281	991	941	923	922
Bulk density *ρ_b_*_2_ [kg/m^3^] Calculated from Equation (6)	280	977	940	935	910
Porosity *p* [%]	85	44	48	49	49

**Table 3:** The obtained values of the thermal parameters of the geopolymer sample and modified gypsum; additionally, calculated statistical parameters.

Statistical Parameters	Thermal Properties of the Geopolymer Sample (GF)			
	λ [W/(m·K)]	Cv [MJ/(m3·K)]	Cp [J/(kg·K)]	a [mm2/s]
Quartile, Q1	0.1140	0.4691	1675	0.2411
Median, M	0.1141	0.4716	1684	0.2418
Quartile, Q3	0.1141	0.4730	1689	0.2432
Interquartile range, IQR = (Q3 − Q1)	0.0001	0.0039	14	0.0021
Higher outlier, HO = Q3 + 1.5·IQR	0.1143	0.4789	1710	0.2464
Lower outlier, LO = Q1 − 1.5·IQR	0.1139	0.4633	1654	0.2380
Average value, $\bar{X}$	0.1141	0.4711	1683	0.2421
Standard deviation, *s*	0.0002	0.0023	8	0.0013
Coefficient of variation, CV [%]	0.14	0.49	0.49	0.53
Upper critical value, UCV	0.1142	0.4735	1691	0.2434
Lower critical value, LCV	0.1139	0.4688	1674	0.2408
**Statistical Parameters**	**Thermal Properties of the Gypsum Sample (G)**			
	***λ* [W/(m·K)]**	***C_v_* [MJ/(m^3^·K)]**	***C_p_* [J/(kg·K)]**	***a* [mm^2^/s]**
Quartile, Q1	0.2887	1.5036	1539	0.1905
Median, M	0.2919	1.5206	1556	0.1919
Quartile, Q3	0.2942	1.5269	1563	0.1936
Interquartile range, IQR = (Q3 − Q1)	0.0055	0.0233	24	0.0031
Higher outlier, HO = Q3 + 1.5·IQR	0.3024	1.5620	1599	0.1984
Lower outlier, LO = Q1 − 1.5·IQR	0.2805	1.4686	1503	0.1858
Average value, $\bar{X}$	0.2917	1.5170	1553	0.1923
Standard deviation, *s*	0.0045	0.0205	21	0.0018
Coefficient of variation, CV [%]	1.55	1.35	1.35	0.92
Upper critical value, UCV	0.2940	1.5281	1563	0.1932
Lower critical value, LCV	0.2895	1.5059	1542	0.1914
**Statistical Parameters**	**Thermal Properties of the Gypsum Sample Modified with Polymer (GP)**			
	***λ* [W/(m·K)]**	***C_v_* [MJ/(m^3^·K)]**	***C_p_* [J/(kg·K)]**	***a* [mm^2^/s]**
Quartile, Q1	0.2478	1.4755	1568	0.1697
Median, M	0.2563	1.4963	1590	0.1716
Quartile, Q3	0.2595	1.5096	1604	0.1729
Interquartile range, IQR = (Q3 − Q1)	0.0117	0.0341	36	0.0032
Higher outlier, HO = Q3 + 1.5·IQR	0.2770	1.5607	1659	0.1776
Lower outlier, LO = Q1 − 1.5·IQR	0.2303	1.4244	1514	0.1649
Average value, $\bar{X}$	0.2556	1.5011	1583	0.1716
Standard deviation, *s*	0.0069	0.0142	33	0.0023
Coefficient of variation, CV [%]	2.70	0.95	2.11	1.36
Upper critical value, UCV	0.2590	1.5088	1603	0.1728
Lower critical value, LCV	0.2522	1.4933	1587	0.1704
**Statistical Parameters**	**Thermal Properties of the Gypsum Sample Modified with Aerogel (GA)**			
	***λ* [W/(m·K)]**	***C_v_* [MJ/(m^3^·K)]**	***C_p_* [J/(kg·K)]**	***a* [mm^2^/s]**
Quartile, Q1	0.2930	1.2775	1390	0.1996
Median, M	0.2959	1.4270	1553	0.2044
Quartile, Q3	0.2998	1.4925	1624	0.2184
Interquartile range, IQR = (Q3 − Q1)	0.0068	0.2150	234	0.0188
Higher outlier, HO = Q3 + 1.5·IQR	0.3101	1.8150	1975	0.2466
Lower outlier, LO = Q1 − 1.5·IQR	0.2828	0.9549	1039	0.1714
Average value, $\bar{X}$	0.2954	1.3994	1523	0.2074
Standard deviation, *s*	0.0072	0.1241	135	0.0095
Coefficient of variation, CV [%]	2.44	8.87	8.87	4.57
Upper critical value, UCV	0.2989	1.4662	1595	0.2123
Lower critical value, LCV	0.2918	1.3326	1450	0.2026
**Statistical Parameters**	**Thermal Properties of the Gypsum Sample Modified with Microspheres (GM)**			
	***λ* [W/(m·K)]**	***C_v_* [MJ/(m^3^·K)]**	***C_p_* [J/(kg·K)]**	***a* [mm^2^/s]**
Quartile, Q1	0.2728	1.4403	1562	0.1855
Median, M	0.2768	1.4549	1578	0.1896
Quartile, Q3	0.2830	1.4858	1611	0.1959
Interquartile range, IQR = (Q3 − Q1)	0.0102	0.0455	49	0.0104
Higher outlier, HO = Q3 + 1.5·IQR	0.2983	1.5540	1685	0.2116
Lower outlier, LO = Q1 − 1.5·IQR	0.2575	1.3721	1488	0.1698
Average value, $\bar{X}$	0.2797	1.4678	1592	0.1930
Standard deviation, *s*	0.0088	0.0310	34	0.0087
Coefficient of variation, CV [%]	3.15	2.11	2.11	4.53
Upper critical value, UCV	0.2840	1.4845	1608	0.1974
Lower critical value, LCV	0.2753	1.4511	1576	0.1885

**Table 4:** The calculated confidence intervals of the measured thermal properties of the building materials.

Tested Sample	Designated Confidence Intervals
GF	P (0.1139 ≤ *λ* ≤ 0.1142) = 0.95
	P (1674 ≤ *C_p_* ≤ 1691) = 0.95
	P (0.2408 ≤ *a* ≤ 0.2434) = 0.95
G	P (0.2895 ≤ *λ* ≤ 0.2940) = 0.95
	P (1542 ≤ *C_p_* ≤ 1563) = 0.95
	P (0.1914 ≤ *a* ≤ 0.1932) = 0.95
GP	P (0.2522 ≤ *λ* ≤ 0.2590) = 0.95
	P (1587 ≤ *C_p_* ≤ 1603) = 0.95
	P (0.1704 ≤ *a* ≤ 0.1728) = 0.95
GA	P (0.2918 ≤ *λ* ≤ 0.2989) = 0.95
	P (1450 ≤ *C_p_* ≤ 1595) = 0.95
	P (0.2026 ≤ *a* ≤ 0.2126) = 0.95
GM	P (0.2753 ≤ *λ* ≤ 0.2840) = 0.95
	P (1576 ≤ *C_p_* ≤ 1608) = 0.95
	P (0.1885 ≤ *a* ≤ 0.194) = 0.95

## Data Availability

Not applicable.
